# Ultrasound characteristics of normal parathyroid glands and analysis of the factors affecting their display

**DOI:** 10.1186/s12880-024-01214-7

**Published:** 2024-02-13

**Authors:** Cuiping Wu, Binyang Zhu, Song Kang, Shiyu Wang, Yingying Liu, Xue Mei, He Zhang, Shuangquan Jiang

**Affiliations:** Department of Ultrasound, The Second Affiliated Hospital of Harbin Medical University, 146 Baojian Road, Harbin City, Heilongjiang Province China

**Keywords:** Parathyroid glands, Ultrasound, Shear wave elastography (SWE), Contrast-enhanced ultrasonography (CEUS), Affecting factors

## Abstract

**Background:**

Parathyroid glands are important endocrine glands, and the identification of normal parathyroid glands is crucial for their protection. The aim of this study is to explore the sonographic characteristics of normal parathyroid glands and analyze the factors affecting their display.

**Methods:**

Seven hundred three subjects who underwent physical examination at our hospital were included. The number, location, size, morphology, echogenicity and blood flow distribution of parathyroid glands were recorded. The ultrasound characteristics and display rate were also summarized. Meanwhile, shear wave elastography was performed in 50 cases to provide the stiffness measurements, and 26 cases received contrast-enhanced ultrasonography for the assessment of microcirculatory perfusion. Furthermore, we analyzed the factors affecting parathyroid display, including basic information of the subjects and ultrasound features of the thyroid.

**Results:**

① A total of 1038 parathyroid glands were detected, among which, 79.29% were hyperechoic, 20.71% were isoechoic, 88.15% were oval-shaped, and 86.71% had blood flow of grade 0-I. ② 81.79% of the subjects had at least one parathyroid gland detected. ③ The Emean, Emax, PI and AUC of the parathyroid glands were significantly lower than those of the adjacent thyroid tissue (*P* < 0.05). ④ The display of normal parathyroid glands was related to BMI, thyroid echogenicity and thyroid volume of the subjects (*P* < 0.05).

**Conclusions:**

Normal parathyroid glands tend to appear as oval-shaped hyperechoic nodules with blood flow of grade 0-I. BMI, thyroid echogenicity and thyroid volume are independent factors affecting the display of parathyroid glands.

**Supplementary Information:**

The online version contains supplementary material available at 10.1186/s12880-024-01214-7.

## Background

Parathyroid glands (PTGs) are vital organs of the endocrine system, which secrete parathyroid hormone(PTH) that regulates the metabolism of calcium and phosphorus in the human body [1]. Irreversible damage to the PTGs or inadvertent removal during thyroid surgery is the leading cause of postoperative hypoparathyroidism [2, 3], which further gives rise to numbness in limbs, muscle spasms and even death in severe cases. The proper identification of PTGs is crucial to their subsequent protection in thyroid surgery.

Currently, intraoperative identification of PTGs is an essential but challenging issue of thyroid surgery, and it relies on the surgeon’s personal experience, nanocarbon or methylene blue injection imaging, intraoperative optical identification, rapid PTH testing, histological identification and imaging examination [4–9]. However, the identification of the PTGs before surgery is mainly based on imaging examination. As for the localization and qualitative diagnosis of parathyroid lesions, CT, MRI and radionuclide imaging have their own merits [10–12]. The sensitivity of CT, MRI and radionuclide imaging for detection of abnormal PTGs is 50–88%,42–90% and 61–92% [13, 14], respectively. The specific sensitivity value varies with the size of the lesion. Nevertheless, there are few articles on the above-mentioned imaging methods to identify normal parathyroid glands, and only one article [15] mentioned that 4D-CT could display the normal parathyroid glands, but the focus is on estimating and comparing the weights of parathyroid adenomas and normal parathyroid glands.

With the continuous improvement of ultrasound technology, high-resolution ultrasound can detect structures as small as 2 mm in diameter [16]. Theoretically, PTGs can be visualized by ultrasound as their diameter is far greater than 2 mm. Furthermore, in some studies, the ultrasound features of normal PTGs were established by biochemical tests [17, 18] (FNA + PTH measurements) or intraoperative findings [19, 20] (made by the surgeon based on experience). The distinctive features of normal PTGs were oval, homogeneous, and mostly hyperechoic nodules, and the accuracy of preoperative ultrasound in identifying normal PTGs was higher than 90% [19]. However, there is a lack of large-scale studies on ultrasound features of normal PTGs. For this reason, we conducted a study of large sample size to investigate the ultrasound characteristics and display rate of normal PTGs. In addition, we further analyzed the factors affecting parathyroid display in an attempt to provide theoretical support for the protection of PTGs, and reduce the incidence of postoperative hypoparathyroidism.

## Methods

### Research subjects

This study was retrospective, though the data used were prospectively collected. A total of 703 adults, including 149 males and 554 females, medically examined at our hospital were selected as subjects, with the age ranging from 18 to 77(44.39 ± 12.19) years old. All subjects were examined by two-dimensional and color Doppler ultrasonography. Among them, 50 subjects underwent shear wave elastography (SWE) and 26 subjects received contrast-enhanced ultrasonography (CEUS). Exclusion criteria included a previous history of thyroid or neck surgery; incomplete ultrasound imaging data and laboratory test results; abnormalities in PTH, calcium ion and phosphorus ion, or imaging findings indicative of parathyroid diseases. This study was approved by the ethics committee of this hospital.

### Instruments

The Preirus and Avius ultrasound scanners (Hitachi, Japan) installed with an L74M transducer (5-13 MHz) were applied to routine ultrasonography. SWE was performed with the SuperSonic Imagine Aixplorer ultrasound scanner (France) and an SL15–4 transducer(4-15 MHz). The CEUS examination carried out using the EPIQ 5C ultrasound scanner (Philips Healthcare, WA) equipped with an L12–3 transducer(3-12 MHz). SonoVue (Bracco SpA, Italy) was used as ultrasound contrast agent (UCA), which consists of microbubbles with a phospholipids monolayer shell enclosing a SF6 gas.

### Research methods

The subjects lay in a supine position with the head gently tilted back to fully expose the neck. The area between the chin, superior sternal fossa, and bilateral common carotid arteries was carefully scanned by taking the thyroid gland as the acoustic window, and the posterior, upper, lower and internal area of thyroid glands were mainly observed. The observed ultrasonic characteristics of PTGs were recorded, including the number, location (with respect to the upper and lower poles of the thyroid and trachea as reference), size, shape, echogenicity (with respect to the normal thyroid), and blood flow distribution based on Adler grade [21]. The images acquired were stored.

After the PTGs and the adjacent thyroid tissues were clearly displayed, the probe was not pressed, and the SWE test was conducted. Specifically, the subjects were asked to hold their breath for a few seconds; the image was frozen when stabilized. The diameter of the sample frame of the area of interest was 2 mm. Then the mean value of elastic modulus (Emean) and the maximum value of elastic modulus (Emax) of the PTGs and adjacent normal thyroid tissues were quantitatively measured. Each section was measured three times and the average was taken.

Prior to the CEUS examination, SonoVue suspension was prepared by the addition of 5 mL of saline solution to a sealed vial. When the PTG and the thyroid gland were displayed in the same view, the subjects kept motionless without holding breath, and the CEUS mode with linear postprocessing function was enabled. The mechanical index was set to be 0.06 to mitigate microbubble destruction, the frame rate was 25 Hz, and the dynamic range was 50d. Subsequently, a bolus of 2 mL UCA was infused through a 20G catheter placed in the median cubital vein. When the UCA was present in the ipsilateral common carotid artery, the dynamic images were saved for at least 1 min. Finally, the built-in software of the ultrasound instrument was employed to plot the time intensity curve. The sampling frame size of the region of interest was 0.5–1.5mm^2^. The peak intensity (PI), the time to peak (TTP) and the area under the curve (AUC) were calculated.

The gender, age, height, weight and the body mass index (BMI) of the subjects were recorded. Besides, the function, echogenicity and volume of their thyroids were documented. The lateral lobe volume of the thyroid was calculated according to the formula V = π/6 × [*L* × *W* × *T*] (*L*, *W* and *T* represent the length, width and thickness of the thyroid gland, respectively), and thyroids with lateral lobe volume > 9.19 ml in male and > 6.19 ml in female were defined as enlargement [22]. The calculation method of PTG volume was the same as that of thyroid glands. The maximum diameter (Dmax) of the thyroid nodules was measured in the subjects with thyroid nodules. All subjects were examined by the same physician with 14 years of experience in thyroid ultrasonography to prevent any human bias and optimize the image quality. Meanwhile, two independent physicians retrospectively analyzed these ultrasound pictures. The final diagnosis was made after consultation if they held different opinions.

### Statistical methods

The data were statistically analyzed using IBM SPSS Statistics 25.0 software. Categorical data was statistically expressed as frequency/percentage, and the chi-square test was adopted for the comparative analysis between groups. Quantitative data that approximately conformed to a normal distribution was statistically represented by ($\overline{x} \pm s$), and the independent samples t-test was adopted for comparison between groups. One-way ANOVA was used to compare the data among multiple groups (Kruskal-Wallis rank sum test was carried out to compare skewed distribution or variance-uneven data). Besides, the Nemenyi method was employed to compare the data between two groups. Influence factor analysis was performed using an unconditional logistic regression analysis model, and variables with a single-factor comparison result of *P* < 0.1 were included in the model (Entry = 0.05, Removal = 0.10). The confidence levels for the hypothesis tests were all determined as α = 0.05.

## Results

### Routine ultrasound characteristics of PTGs

**Fig. 1:**
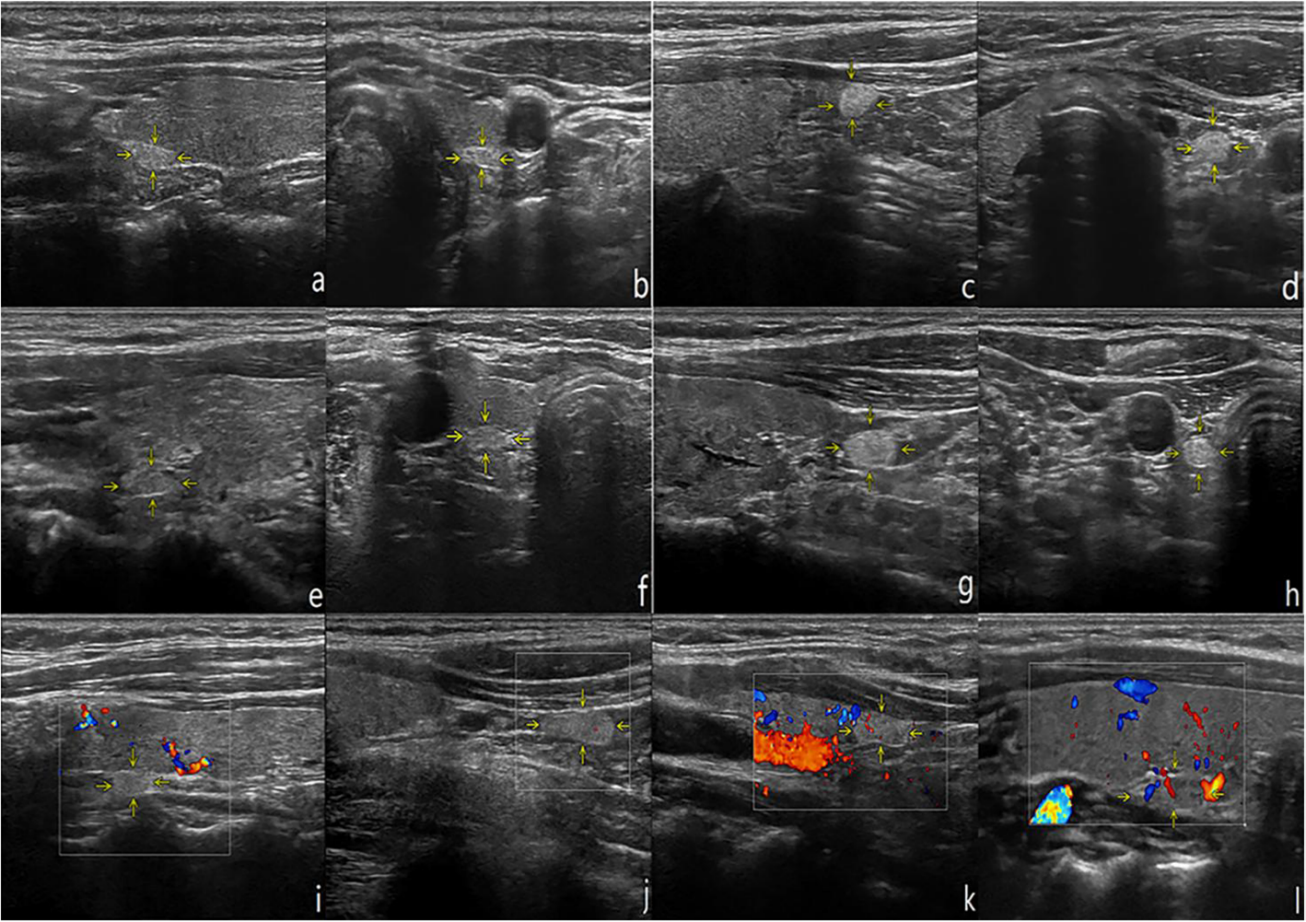
Routine Ultrasound Characteristics of normal PTGs. **a**-**d**: Longitudinal and transverse views of the left superior and left inferior PTGs, respectively. **e**-**h**: Longitudinal and transverse views of the right superior and right inferior PTGs, respectively. **i**-**l**: Internal blood flow signal of PTGs, in the order of grade 0 to grade III

A total of 1038 PTGs were detected from 703 subjects, all of which were small nodules with clear boundaries. Their echogenicity, morphology, location and blood flow distribution are shown in Table 1 and Fig. 1. The difference in PTG volume between men and women was statistically significant (*P* < 0.001), while there was no significant difference in parathyroid volume with respect to age (*P* = 0.488) (Table 2).

**Table 1:** Routine Ultrasound Characteristics of Normal PTGs

	Echo		Shape				Relative Location to Thyroid Capsule			CDFI			
	Hyperechogenicity	Isoechogenicity	Oval shape	Round-like shape	Teardrop shape	Irregular shape	Outside the capsule	Part of the capsule	Inside the capsule	Grade 0	Grade I	Grade II	Grade III
Number	823	215	915	74	23	26	1009	16	13	512	388	104	34
Proportion(%)	79.29	20.71	88.15	7.13	2.22	2.50	97.21	1.54	1.25	49.33	37.38	10	3.28

**Table 2:** Size of Normal PTGs (x±s)

	Total	Gender		*t*-value	*P*-value	Age					*t*-value	*P*-value
		Male	Female			18-29	30-39	40-49	50-59	≥60		
Length(mm)	6.6±1.4	6.7±1.5	6.6±1.4	1.586	0.113	6.5±1.6	6.6±1.4	6.6±1.3	6.6±1.5	6.7±1.4	2.516	0.113
Width(mm)	4.8±1.1	4.9±1.2	4.7±1.1	2.181	0.020	4.7±1.0	4.8±1.0	4.8±1.1	4.8±1.1	4.8±1.1	5.477	0.019
Thickness(mm)	3.6±0.8	3.9±0.9	3.5±0.8	4.845	<0.001	3.5±0.7	3.5±0.8	3.6±0.9	3.6±0.9	3.8±0.8	11.037	0.026
Volume(mm^3^)	62.1±32.3	69.8±36.9	60.0±30.6	3.975	<0.001	59.4±35.2	60.2±28.0	62.1±31.3	63.2±35.0	65.6±32.7	3.432	0.488

### Location distribution of PTGs

**Fig. 2:**
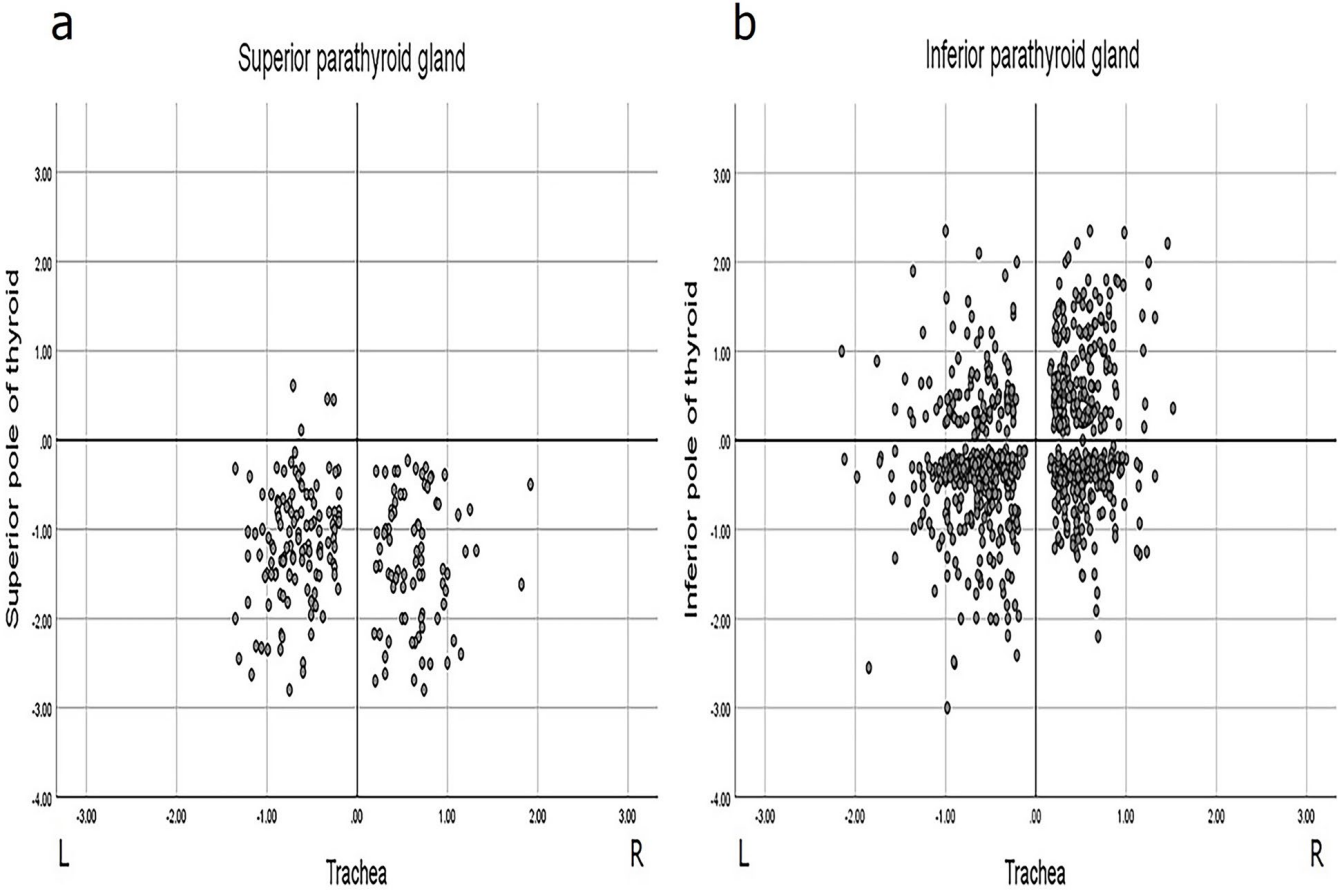
Distribution of PTGs Location. **a**: Distribution of the location of the superior PTGs. **b**: Distribution of the location of the inferior PTGs. The horizontal coordinate represents the distance of the PTG from the lateral margin of the trachea, while the vertical coordinate represents the distance of the PTG from the superior pole (or inferior pole)

Among 1038 PTGs, 206 (19.85%) were superior PTGs, including 123 (11.85%) on the left side and 83 (8.00%) on the right side; 832 (80.15%) were inferior PTGs, including 442 (42.58%) on the left side and 390 (37.57%) on the right side. The distance from the left superior PTG to the superior thyroid pole and the trachea was 0.11–2.80 (1.18 ± 0.60) cm and 0.20–1.35 (0.67 ± 0.29) cm, respectively. The right superior PTG was 0.23–2.80 (1.36 ± 0.71) cm away from the superior thyroid pole and 0.19–1.92 (0.64 ± 0.34) cm away from the trachea. The left and right inferior PTGs were 0.06–3.50 (0.60 ± 0.50) cm and 0.07–2.35 (0.67 ± 0.48) cm away from the inferior thyroid pole, 0.12–2.50 (0.64 ± 0.35) cm and 0.16–2.46 (0.53 ± 0.28) cm away from the trachea, respectively. By taking the intersection of the horizontal line of the superior or inferior pole of the thyroid gland in the left and right lobes and the lateral margin of the trachea were determined as the reference points, and the location distribution of PTGs was plotted, as shown in Fig. 2.

### SWE and CEUS characteristics of PTGs

**Fig. 3:**
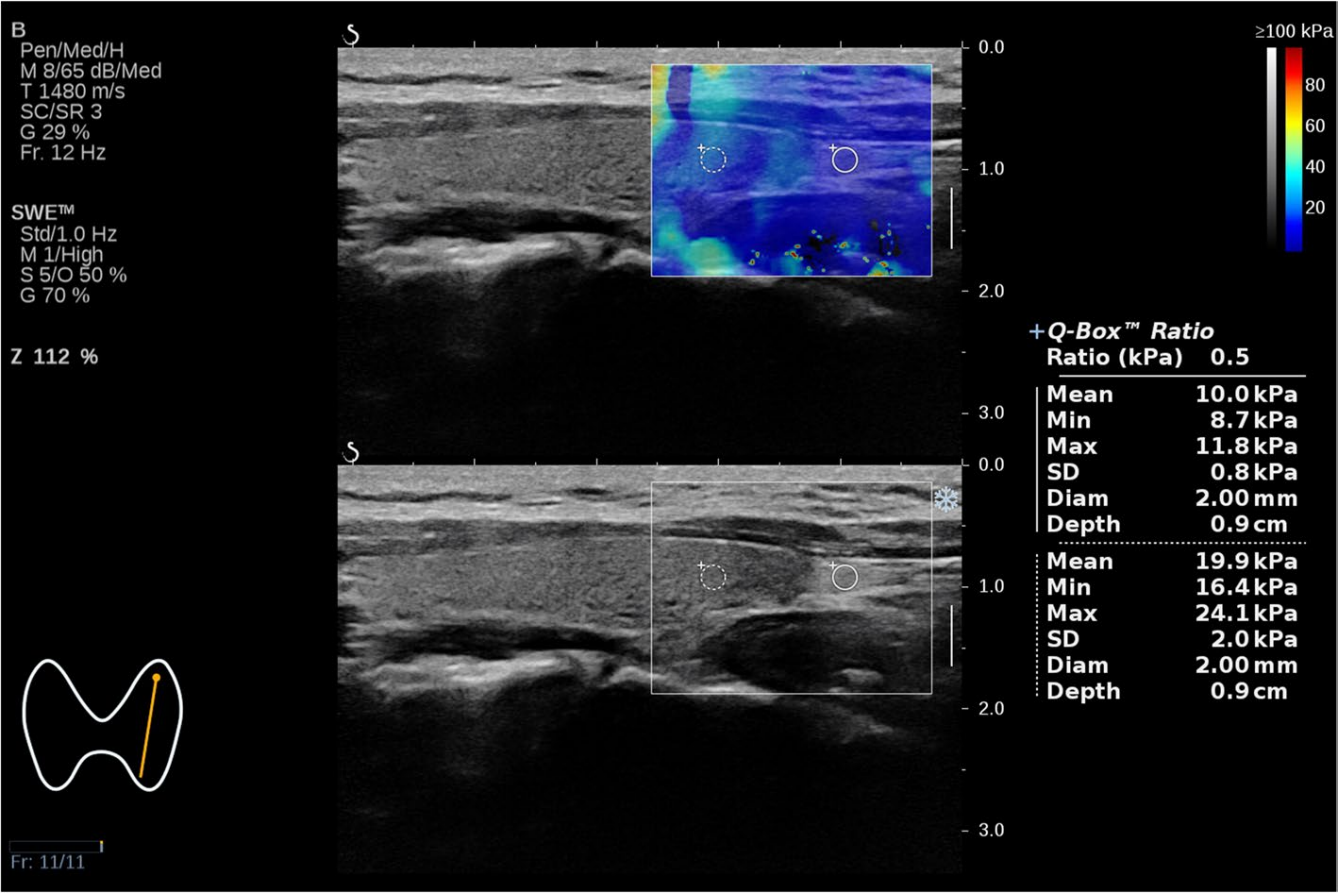
SWE Image of PTG, the left circle of sampling frame represented thyroid tissue, the right circle of sampling frame represented the PTG tissue

A total of 78 PTGs were detected from 50 subjects who underwent SWE. The PTG was rendered predominantly blue in the SWE image (Fig. 3). The difference in the Emax and Emean between PTGs and adjacent thyroid gland was statistically significant (*P* < 0.001) (Table 3).

**Table 3:** Comparative analysis of SWE and CEUS parameters between PTG and Thyroid

	PTG	thyroid	*t*-value	*P*-value
Emean (kPa)	14.95 ± 7.10	24.28 ± 8.85	−11.688	< 0.001
Emax (kPa)	19.50 ± 8.55	27.61 ± 9.71	−8.615	< 0.001
TTP (s)	5.10 ± 1.88	5.12 ± 2.00	−0.197	0.846
PI (dB)	12.02 ± 2.56	9.06 ± 2.50	5.791	< 0.001
AUC (dB sec)	403.46 ± 155.95	280.16 ± 105.00	4.435	< 0.001

**Fig. 4:**
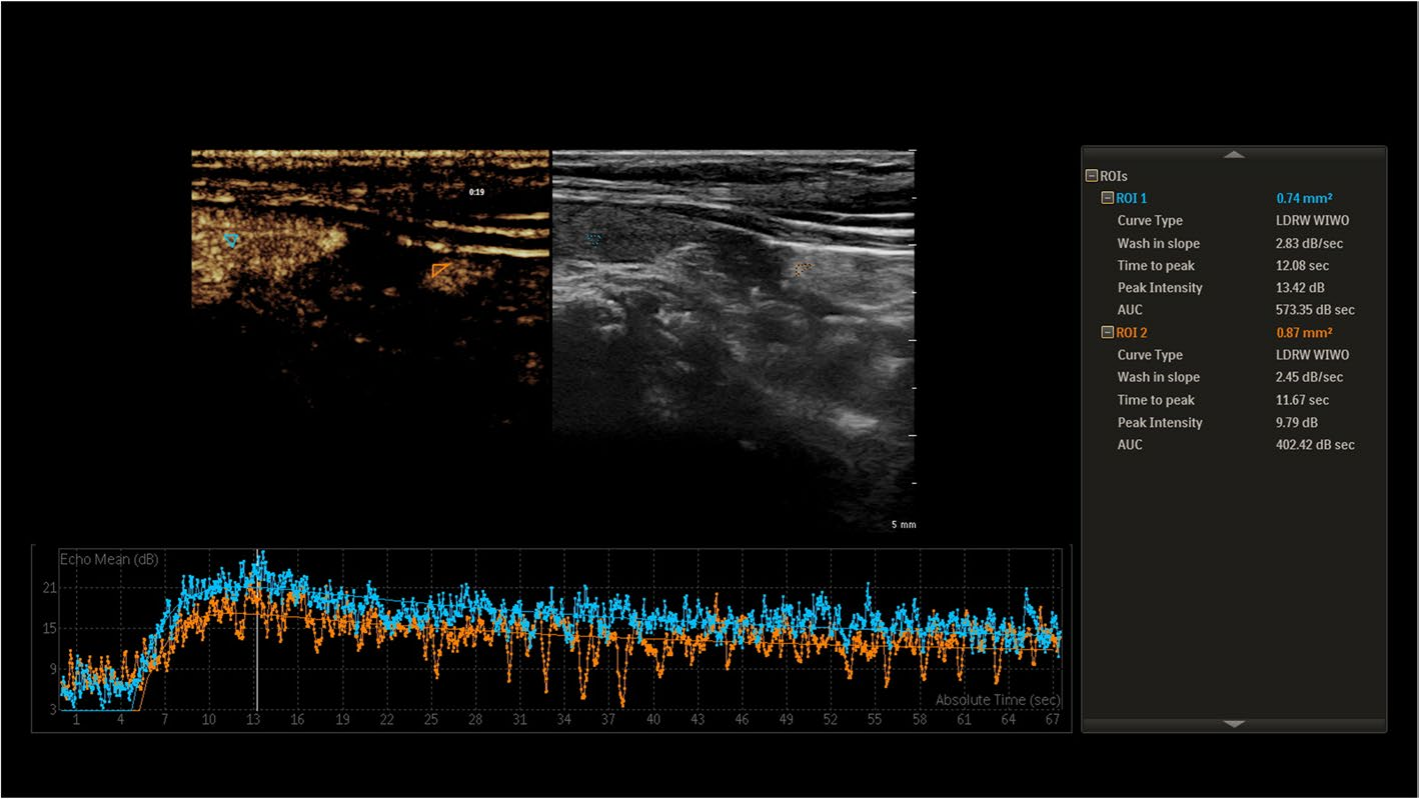
CEUS Performance of PTG, the blue line is the time-intensity curve of the thyroid, the yellow line is the time-intensity curve of the PTG

A total of 28 PTGs were detected in 26 subjects who took the CEUS examination, with a manifestation of uniform and moderate enhancement (Fig. 4). Statistically significant difference was observed in the PI and AUC between PTGs and adjacent thyroid gland (*P* < 0.001), but the difference in TTP was not statistically significant (*P* = 0.846) (Table 3).

### Display rate of Normal PTGs by ultrasonography and its influencing factors

Among 703 subjects, 25(3.56%) had 4 PTGs displayed, 73(10.38%) had 3 PTGs displayed, 242(34.42%) had 2 PTGs displayed, and 235(33.43%) had 1 PTG displayed. 128(18.21%) subjects failed to have any PTGs detected. At least one PTG was displayed in 81.79% of these subjects. Univariate analysis showed that the display of normal PTGs was not significantly related to the age and gender of the subjects (*P* > 0.05), but associated with the BMI, thyroid echogenicity, thyroid function, thyroid volume and Dmax of thyroid nodules of the subject (*P* < 0.10) (Table 4). Nevertheless, multivariate analysis indicated that BMI, thyroid echogenicity and thyroid volume were independent influencing factors of the display of normal PTGs (*P* < 0.05) (refer to Table 5).

**Table 4:** Comparative analysis of demographic information and clinical characteristics

	Display Group (*n* = 575)	Non-display Group (*n* = 128)	*t/χ*^*2*^-value	*P-value*
Age (*n*,%)			6.082	0.193
18–29	66(11.5)	20(15.6)		
30–39	132(23.0)	30(23.4)		
40–49	158(27.4)	33(25.9)		
50–59	161(28.0)	26(20.3)		
≥ 60	58(10.1)	19(14.8)		
Gender(*n*,%)			2.148	0.143
Male	128(22.3)	21(16.4)		
Female	447(77.7)	107(83.6)		
BMI^a^, kg/m^2^			16.416	0.001
< 18.5	20(3.5)	14(10.9)		
18.5–24.0	279(48.5)	62(48.5)		
24–28	187(32.5)	42(32.8)		
≥ 28	89(15.5)	10(7.8)		
Thyroid Echo			26.766	< 0.001
Normal	493(85.7)	85(66.4)		
Abnormal	82(14.3)	43(33.6)		
Thyroid Function^b^			2.896	0.089
Normal	441(76.7)	89(69.5)		
Abnormal	134(23.3)	39(30.5)		
Thyroid Volume			11.001	0.001
Normal	402(69.9)	70(54.7)		
Enlargement	173(30.1)	58(45.3)		
Dmax^c^			2.870	0.057
None	146(25.4)	32(25.0)		
≤ 2,cm	372(64.7)	74(57.8)		
> 2,cm	57(9.9)	22(17.2)		

^a^The BMI was graded according to the Chinese standard. ^b^If the thyroid function index is not within the range of reference value, it is defined as abnormal function. ^c^The maximum diameter of a thyroid nodule

**Table 5:** Multiple-factor analysis affecting the display of PTGs

	*β*	*SE*	*OR*	*OR 95%CI*	*P-value*
BMI < 18.5					0.002
18.5 ≤ BMI < 24.0	1.174	0.392	3.235	1.499–6.981	0.003
24.0 ≤ BMI < 28.0	1.267	0.408	3.549	1.597–7.889	0.002
BMI ≥ 28.0	1.849	0.500	6.354	2.386–16.919	< 0.001
Thyroid Echo	0.996	0.229	2.706	1.729–4.236	< 0.001
Thyroid Volume	0.632	0.209	1.881	1.248–2.832	0.003

## Discussion

In the present study, we evaluated the ultrasound characteristics and display rate of normal PTGs, and analyzed the factors affecting parathyroid display. It was found that normal PTGs were mostly homogeneously hyperechoic or isoechoic oval nodules with well-defined boundaries.

The investigation on ultrasound characteristics of normal PTGs is of great clinical significance, contributing to the preoperative localization and better intraoperative protection. However, there is a long-standing controversy about whether normal PTGs can be showed on ultrasound. It was suggested in a study [23] that normal PTGs were tiny in size and hardly differentiated from adjacent tissues in the acoustic impedance, and thus they could not be displayed properly. However, in theory, high-resolution ultrasound can clearly display normal PTGs. Lee [24] concluded that normal PTGs could be shown on high frequency ultrasound images, mostly manifested as hypoechoic nodules. Piciucchi [25] indicated that normal PTGs were presented as isoechoic nodules. Nevertheless, the intraoperative ultrasonographic study by Xia [20] suggested that normal PTGs were clearly visualized as hyperechoic nodules. According to Chen [19], ultrasound had an excellent value in recognizing normal PTGs, which primarily showed oval-shaped hyperechoic nodules. Therefore, we conducted this study based on previous articles.

In this study, 79.3% of PTGs were hyperechoic and 20.7% were isoechoic. The echo of PTGs was directly related to their internal composition. Adipose tissues are enriched in PTGs because of the presence of an enormous number of fat particles in the cytoplasm of the principal cells and the stroma. Therefore, normal PTGs were moderately hyperechoic, while changes in the fat content of PTGs might influence their echo [26].

Normal PTGs have various morphologies, among which, elliptical shape is the commonest, accounting for 88.15% of all PTGs detected in the subjects enrolled in this study. This finding was consistent with that of Shou [27]. The mean size of PTGs measured by ultrasound in this study [(6.6 ± 1.4) mm in length, (4.8 ± 1.1) mm in width, and (3.6 ± 0.8) mm in thickness] was close to determined by clinically anatomical and embryological findings (5.0–6.0 mm × 3.0 mm × 1-2 mm) [28].

Relatively few studies have investigated SWE and CEUS of the normal PTGs. According to the present study results, Young’s modulus values of the PTG measured by SWE were lower than those of adjacent thyroid tissues, indicating that the PTG is softer due to rich fat content in the cytoplasm and stroma [26]. By contrast, the thyroid gland consists primarily of thyroid follicular cells and parafollicular cells with connective tissue between follicles [29]. As a result, the thyroid gland has a stiffer texture than the PTG. Moreover, PI and AUC of PTGs evaluated by CEUS were lower than those of adjacent thyroid tissues. It may be attributed to the presence of plenty of porous capillaries between follicular cells within the thyroid gland [29], while the blood supply of the PTG are terminal vessels. Correspondingly, the normal parathyroid blood flow in this study was mostly of grade 0 (no blood flow signal) and grade I (punctate blood flow signal) on CDFI.

A total of 1038 PTGs were detected by ultrasonography in 703 subjects, which was probably ascribed to the limitations of ultrasonography and ectopic PTGs. Among these PTGs, 19.85% were superior PTGs and 80.15% were inferior PTGs. The display rate of inferior PTGs by ultrasound was significantly higher than that of superior PTGs. This result differed from the intraoperative exploration of the PTGs by the surgeons. The superior PTGs were more easily identified during surgery and mostly located near the junction of the middle and upper 1/3 posterior to the lateral lobe of the thyroid. The reason for this phenomenon is that on account of the relatively short migration distance of the superior PTGs during embryonic development, their anatomic location tends to be fixed and changes slightly. The low display rate of superior PTGs in preoperative examination may be attributed to the high susceptibility of soft PTGs to distortion and shrinkage under the compression of the relatively dense adjacent tissues, making them less accessible on ultrasonography. The inferior PTG migrates a long distance during embryonic development leading to a large alteration in its location, mostly in the region between the inferior pole of the thyroid gland and the thymus [30]. Some inferior PTGs hidden in fatty tissues are difficult for surgeons to detect, but they can be easily displayed on ultrasonography owing to their distinct difference from adjacent tissues in acoustic impedance. In addition, these PTG tissues around the lower pole of the thyroid are so loose that they are hardly squeezed by adjacent tissues such as the trachea. Preoperative ultrasound and intraoperative exploration complement each other and help preserve the PTGs in situ.

Nearly one-fifth of the subjects in this study still failed to have at least one PTG displayed. Through further analysis, it was revealed that independent factors affecting the display of normal PTGs were BMI classification, thyroid echogenicity, and thyroid volume. With the increase of BMI, the PTG display rate increased. It is probably because an increase in the fat content in PTGs makes it easier for them to be identified and displayed [26]. The change of thyroid echogenicity, such as Hashimoto’s thyroiditis with fibrous cords, will make the acoustic interface of the thyroid and its surrounding tissues more complex and disordered, rendering the discrimination of the PTGs from adjacent tissues more difficult. Moreover, when the size of the thyroid gland increases, the PTGs are easily deformed by pressure due to their soft texture, thus resulting in a decrease in the display rate. Given the significance of parathyroid glands for the body’s metabolism, it is hoped that multimodality imaging [31, 32] will be used to identify normal parathyroid glands in future research and improve the display rate of normal parathyroid glands.

The study of Song [33] suggested that the preservation of at least one PTG with an intact blood supply during total thyroidectomy was sufficient to prevent the development of permanent hypoparathyroidism. In the present study 81.79% of the subjects had at least one PTG displayed. Therefore, preoperative ultrasound localization is conducive to the exploration and protection of PTGs, especially the PTGs embedded in fatty tissues or far from thyroid tissues. It reduces unnecessary intraoperative manipulations, thus improving the efficiency of surgery and better preventing the occurrence of postoperative hypoparathyroidism.

One of the contributions of this study is that we provide the ultrasound characteristics of normal PTGs from the largest cohort of healthy people to date. This is the first study to investigate SWE and CEUS of normal PTGs. However, there are also some limitations in our investigation. First, ultrasonography is subjective in essence, and the display of the PTGs depends on the experience and scanning skills of the sonographer. Second, there is a lack of refinement and quantification of the abnormalities in thyroid function and echogenicity in this study. Third, due to the lack of histological findings, there is a certain quantity of false negatives or false positives in ultrasound-determined PTGs.

## Conclusion

In summary, normal PTGs can be displayed by high-frequency ultrasonography, mostly presenting like homogeneously hyperechoic or isoechoic oval nodules with clear boundaries, and showing punctate or no blood flow signal on CDFI. BMI, thyroid echogenicity and thyroid volume are independent influencing factors for the display of PTGs. The precise identification and localization of the normal PTGs by ultrasound facilitates the intraoperative protection of the PTGs and their differentiation from periparathyroidal lesions.

### Supplementary Information

**Additional file 1.**

## Data Availability

All data generated or analysed during this study are included in this published article [and its supplementary information files].

## Abbreviations

PTGs: Parathyroid glands
PTH: Parathyroid hormone
SWE: Shear wave elastography
CEUS: Contrast-enhanced ultrasonography
UCA: Ultrasound contrast agent
Emean: The mean value of elastic modulus
Emax: The maximum value of elastic modulus
PI: Peak intensity
TTP: Time to peak
AUC: Area under the curve
BMI: Body mass index
Dmax: Maximum diameter
